# Personal

**Published:** 1900-08

**Authors:** 


### PERSONAL.

Dr. A. M. Phelps, of New York City, president of the Medical Society of the State of New York, attended the annual meeting of the Oregon State Medical Society, held at Portland, June 26 and 27, 1900. Dr. Phelps gave up an attractive trip elsewhere that he had in contemplation, for the purpose of accepting the invitation of the Oregon Society. He read three papers, or demonstrated three subjects, relating to orthopedic surgery. The meeting was the most successful in the history of the society.

---

Dr. Leonard B. Almy, of Norwich, was elected president of the Connecticut State Medical Society at its annual meeting, held at New Haven, May 23 and 24, 1900. Dr. Almy served as chief surgeon of the 2d Division of the 2d Army Corps, during the Spanish-American war, and was also in charge of a section of the general hospital at Montauk Point until the close of the war. Dr. Almy visited Buffalo early in July and was the guest of his father, A. H. Almy, Esq., of 930 Main Street.

---

Dr. Robert K. Grove, of Buffalo, who for the past eighteen months has been prosecuting his studies in Europe and in Dr. Knapp's Hospital, New York City, announces that he is now associated with Dr. Benjamin H. Grove, at 334 Pearl Street, near Huron Street, in the treatment of diseases of the eye, ear, nose and throat. Consultation hours: 8 a.m. to 1 p.m.; 6.30 to 7.30 p.m. Sundays: 9 to 10 a.m.; 12.30 to 1.30 p.m. Other hours by appointment.

---

Dr. R. B. Granger, of New York, who was for a number of years in charge of D. Appleton & Co.'s medical publications, announces to his medical friends and acquaintances that he has established business relations with the American Plasmon Syndicate, limited, of New York. Dr. Granger's many friends in the medical profession will be pleased to learn of his new enterprise and bright prospects.

---

Dr. A. Vander Veer, of Albany, N.Y., professor of abdominal surgery and dean of the faculty of Albany Medical College, and a regent of the University of the State of New York, has recently been appointed a member of the consulting board of the New York State Hospital for the care of crippled and deformed children.

---

Dr. Arthur MacDonald, specialist in the United States Bureau of Education at Washington, and Dr. Ella B. Everett, resident physician at Woman's Hospital, Philadelphia, are announced as engaged to be married. We believe the nuptials will be celebrated at an early day.

---

Dr. William F. Beck, U. of B. '93, of Vermillion, O., paid a brief visit to Buffalo during the latter part of July. Dr. Beck took his wife from this city, and the death of Mrs. Beck's father was the occasion of a sad errand of duty by Dr. and Mrs. Beck at the time mentioned.

---

Dr. Marie Louise Benoit, U. of B., '96, until recently a medical interne at the Craig Colony for Epileptics, has been promoted from the civil service list to the position of resident physician at the State Custodial Asylum for Feeble-minded Women, at Newark, N. Y.

---

Hon. Henry W. Hill, of Buffalo, N.Y., who has represented the second assembly district of Erie County in the legislature for several years, received the degree of LL. D., at the last annual commencement of the University of Vermont.

---

Dr. M. E. Fisher, U. of B. '94, of Yorkshire, N. Y., was nominated for the Assembly at the recent convention held in the 2d Assembly District of Cattaraugus County. It took eighty-five ballots to do the work. Dr. Fisher is to be congratulated on the result.

Dr. Victor C. Pedersen, of New York, has been appointed assistant demonstrator of anatomy at the College of Physicians and Surgeons, and assistant surgeon to the out-patient department of Roosevelt Hospital. Dr. Pedersen is a brother of Dr. James Pedersen, who is adjunct professor of venereal and genito-urinary surgery at the Post-Graduate Medical School.

---

Dr. Irving P. Lyon, of Buffalo, announces the change of his residence and office from No. 75 Niagara Street to 531 Franklin Street. Hours: 8 to 9.30 a.m.; 2 to 4 p.m. Telephone, Tupper 614.

---

Dr. W. Scott Renner, of Buffalo, was elected vice-president of the American Laryngological, Rhinological and Otological Association at its last annual meeting, held at Philadelphia.

---

Dr. R. T. Rolph, of Dunkirk, has been appointed resident physician in the hotel and sanitarium at Castle Creek, Hot Springs, Arizona.

